# Predictive significance of DNA damage and repair biomarkers in triple-negative breast cancer patients treated with neoadjuvant chemotherapy: An exploratory analysis

**DOI:** 10.18632/oncotarget.6001

**Published:** 2015-10-17

**Authors:** Patrizia Vici, Anna Di Benedetto, Cristiana Ercolani, Laura Pizzuti, Luigi Di Lauro, Domenico Sergi, Francesca Sperati, Irene Terrenato, Rosanna Dattilo, Claudio Botti, Alessandra Fabi, Maria Teresa Ramieri, Lucia Mentuccia, Camilla Marinelli, Laura Iezzi, Teresa Gamucci, Clara Natoli, Ilio Vitale, Maddalena Barba, Marcella Mottolese, Ruggero De Maria, Marcello Maugeri-Saccà

**Affiliations:** ^1^ Division of Medical Oncology B, “Regina Elena” National Cancer Institute, Rome, Italy; ^2^ Department of Pathology, “Regina Elena” National Cancer Institute, Rome, Italy; ^3^ Biostatistics-Scientific Direction, “Regina Elena” National Cancer Institute, Rome, Italy; ^4^ Scientific Direction, “Regina Elena” National Cancer Institute, Rome, Italy; ^5^ Department of Surgery, “Regina Elena” National Cancer Institute, Rome, Italy; ^6^ Division of Medical Oncology A, “Regina Elena” National Cancer Institute, Rome, Italy; ^7^ Division of Pathology, ASL Frosinone, Frosinone, Italy; ^8^ Medical Oncology Unit, ASL Frosinone, Frosinone, Italy; ^9^ Division of Pathology, “SS. Annunziata Hospital”, Chieti, Italy; ^10^ Department of Experimental and Clinical Sciences, University “G. d'Annunzio”, Chieti, Italy; ^11^ Department of Biology, University of Rome “Tor Vergata”, Rome, Italy

**Keywords:** DNA damage and repair, triple-negative breast cancer, pathological complete response

## Abstract

Response of cancer cells to chemotherapy-induced DNA damage is regulated by the ATM-Chk2 and ATR-Chk1 pathways. We investigated the association between phosphorylated H2AX (γ-H2AX), a marker of DNA double-strand breaks that trigger the ATM-Chk2 cascade, and phosphorylated Chk1 (pChk1), with pathological complete response (pCR) in triple-negative breast cancer (TNBC) patients treated with neoadjuvant chemotherapy. γ-H2AX and pChk1 were retrospectively assessed by immunohistochemistry in a series of pretreatment biopsies related to 66 patients. In fifty-three tumors hormone receptor status was negative in both the diagnostic biopsies and residual cancers, whereas in 13 cases there was a slight hormone receptor expression that changed after chemotherapy. Internal validation was carried out. In the entire cohort elevated levels of γ-H2AX, but not pChk1, were associated with reduced pCR rate (*p* = 0.009). The association tested significant in both uni- and multivariate logistic regression models (OR 4.51, 95% CI: 1.39–14.66, *p* = 0.012, and OR 5.07, 95% CI: 1.28–20.09, *p* = 0.021, respectively). Internal validation supported the predictive value of the model. The predictive ability of γ-H2AX was further confirmed in the multivariate model after exclusion of tumors that underwent changes in hormone receptor status during chemotherapy (OR 7.07, 95% CI: 1.39–36.02, *p* = 0.018). Finally, in residual diseases a significant decrease of γ-H2AX levels was observed (*p* < 0.001). Overall, γ-H2AX showed ability to predict pCR in TNBC and deserves larger, prospective studies.

## INTRODUCTION

Triple-negative breast cancer (TNBC) accounts for approximately 20% of all breast cancer (BC) cases, and represents the most aggressive BC subtype [1]. Neoadjuvant chemotherapy (NACT) was historically delivered with the aim to shrink unresectable tumors or increase the rate of breast-conserving surgery for operable tumors [2]. Due to evidence linking pathological complete response (pCR) to improved survival outcomes [3], the neoadjuvant setting is increasingly exploited as a platform in the search for predictive biomarkers [4].

Escape from chemotherapy-induced death stimuli is a multifaceted phenomenon mediated by both cancer cell-intrinsic and -extrinsic factors [5]. Cancer cells “hijack” physiological mechanisms to endure perturbations arising or induced in their eco-system, such as exposure to chemotherapy. The pronounced ability to protect the genome is one of the best preclinically described way through which cancer cells survive chemotherapy [6]. Safeguarding genome integrity and preventing the accumulation of harmful mutations is a complex task, whose accomplishment requires a tight cooperation between a number of pathways. This intricate network, overall defined as the DNA damage repair (DDR), schematically operates through the coordinated activity of three major signals [7]: cell cycle checkpoints that halt the progression of the cell cycle when DNA damage is sensed, DNA repair mechanisms that remove DNA lesions, and apoptotic pathways that eliminate cells whose genetic lesions cannot be repaired [7]. Over the past decade, the complexity of the DDR has been the focus of intense preclinical investigations, and nowadays we have a fairly detailed picture of the molecular events that are triggered in cancer cells challenged with chemotherapy [8]. Despite these achievements, from a clinical perspective the analyses of the DDR have historically been confined to a handful of distal effectors acting in the context of specific repair avenues, and they have been overall inconclusive [9]. More recently, novel biomarkers such as RAD51 and the so-called genomic scars, which presumably reflect the underlying state of DNA repair, were proposed, renewing the enthusiasm surrounding the clinical development of DDR-associated endpoints to foresee the efficacy of chemotherapy [10–13].

Phosphorylated (Ser139) H2A Histone Family Member X (γ-H2AX) is an established marker of DNA double-strand breaks (DSBs) [14]. When these lesions occur, the Ataxia-Telangiectasia Mutated (ATM)-Checkpoint Kinase 2 (Chk2) pathway is activated and orchestrates DNA repair [15]. In a treatment-naïve background, elevated γ-H2AX levels might mirror a strategy, namely the activation of the ATM-Chk2 pathway, cancer cells evolved to tolerate endogenous DNA damages arising upon oncogene-induced replication stress [16]. We reasoned that this adaption to deal with replicative stress concomitantly feeds therapeutic resistance.

A second key DDR pathway is the Ataxia Telangiectasia and Rad3-related protein (ATR)-Checkpoint kinase 1 (Chk1)-Wee1-like protein kinase (Wee1) signal [17]. The ATR-Chk1-Wee1 axis, that extensively cooperates with the ATM-Chk2 pathway, is mainly activated by stretched of single-stranded DNAs and governs G_2_/M transition. By arresting the cell cycle, the ATR-Chk1-Wee1 signal avoids that damaged cells embark into a fatal mitosis.

Given that γ-H2AX and Chk1 operate in the context of major molecular routes deputed to initiate the DDR, their expression might mirror an underlying chemoresistant phenotype. To test this hypothesis, γ-H2AX and phosphorylated Chk1(pChk1) were evaluated by immunohistochemistry (IHC) in pretreatment biopsies related to TNBC patients treated with anthracycline-taxane-based NACT, and their expression analyzed for a potential association with pCR.

## RESULTS

**Figure 1:**
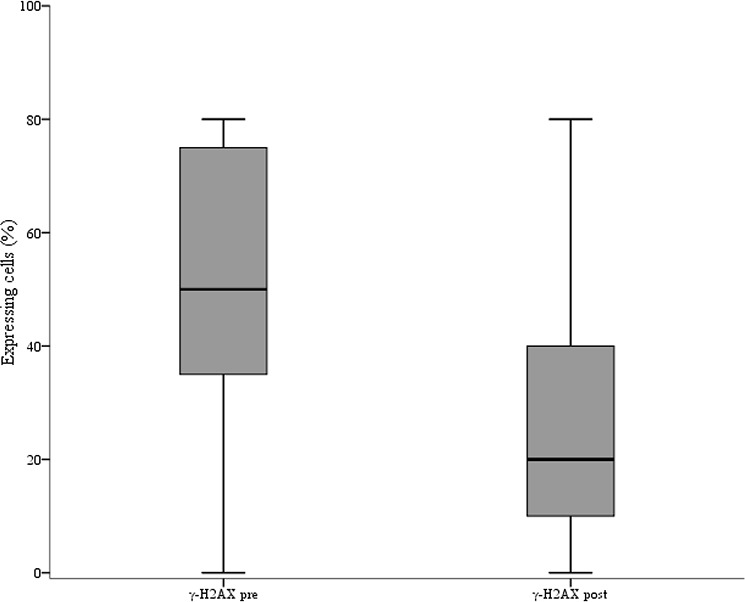
Box plot showing the distribution of γ-H2AX values in pre and post-neoadjuvant chemotherapy samples The figure shows the median values (horizontal bars within boxes), 25th and 75th percentile (lower and upper horizontal lines of the boxes), and minimum and maximum values (lower and upper horizontal bars outside the boxes).

**Figure 2:**
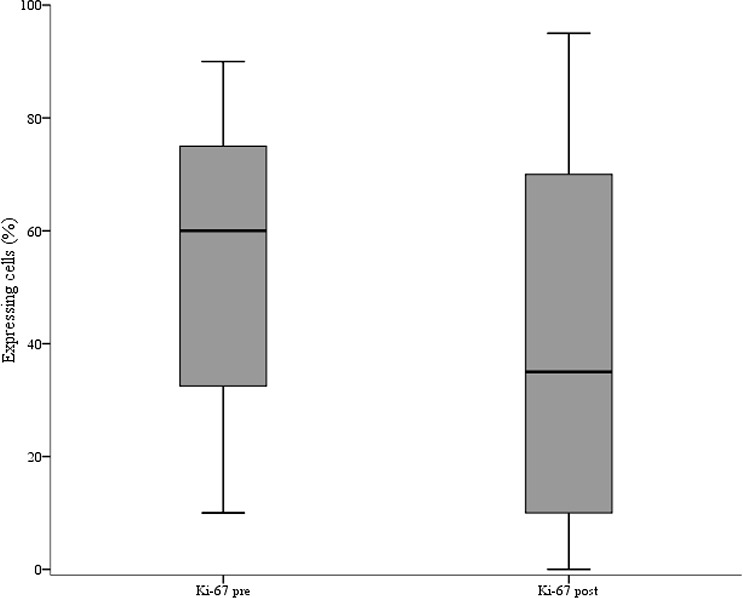
Box plot of the distribution of Ki-67 values in pre and post-neoadjuvant chemotherapy samples The figure shows the median values (horizontal bars within boxes), 25th and 75th percentile (lower and upper horizontal lines of the boxes), and minimum and maximum values (lower and upper horizontal bars outside the boxes).

Baseline characteristics and treatment outcome of the 66 patients included in the present study are illustrated in Table 1. As showed in Table 2, we observed a significant association between elevated γ-H2AX levels and reduced pCR rate (*p* = 0.009). In the γ-H2AX^low^ group we recorded 14 pCRs (43.8%) and 18 (56.2%) residual diseases, whereas in the γ-H2AX^high^ group we observed 5 pCR (14.7%) and 29 residual diseases (85.3%). Conversely, pChk1 expression did not appear associated with pCR (*p* = 0.085), (Table 2). The predictive ability of γ-H2AX levels was observed in the univariate logistic regression model (γ-H2AX^high^ vs γ-H2AX^low^: Odds Ratio (OR) 4.51, 95% Confidence Interval (CI): 1.39–14.66, *p* = 0.012) (Table 3), and maintained in the multivariate model (γ-H2AX^high^ vs γ-H2AX^low^: OR 5.07, 95% CI: 1.28–20.09, *p* = 0.021) (Table 3). The consistency of the multivariate model was supported by internal validation envisioning a re-sampling without replacement procedure. Median Cohen's Kappa coefficient was 0.492 (moderate agreement), and the replication rate for γ-H2AX was 67%. Sensitivity analysis carried out by removing 13 patients whose tumors changed hormone receptor status during NACT further confirmed the predictive ability of γ-H2AX^high^ (univariate and multivariate logistic regression models: γ-H2AX^high^ vs γ-H2AX^low^: OR 4.71, 95% CI: 1.26–17.66, *p* = 0.021; and OR 7.07, 95% CI: 1.39–36.02, *p* = 0.018, respectively) (Table 4). A suggestion for a predictive role of pChk1 stemmed from the 13 tumors that switched hormone receptor expression. In this small subset, we observed 9 residual diseases and 1 pCR in pChk1^pos^ tumors, whereas all the three patients with pChk1^neg^ tumors experienced a pCR (*p* = 0.014, data available upon request). Finally, analysis of matched pre- and post-treatment tissues showed a significant reduction of both γ-H2AX and Ki-67 expression in residual disease (*p* < 0.001 and *p* = 0.012 in Figure 1 and Figure 2, respectively).

**Table 1:** Baseline characteristics and treatment outcome of TNBC patients treated with neoadjuvant chemotherapy (*N* = 66)

Characteristics	*N* (%)
**Age at diagnosis** Mean ± SD Median (min-max)[IQrange]	49.6 ± 11.4 48.4 (25.6–76.6) [44.0–57.6]
**Stage** II III	23 (34.8) 43 (65.2)
**Ki-67** Mean ± SD Median (min-max)[IQrange]	57.0 ± 25.0 60 (10–90) [40–80]
**Grade** 1–2 3	26 (39.4) 40 (60.6)
**Chemotherapy** Sequential Concomitant	56 (84.8) 10 (15.2)
**Hormone receptor change** No Yes	53 (80.3) 13 (19.7)
**Pathological complete response** Yes No	19 (28.8) 47 (71.2)

**Table 2:** Association between biomarkers of DNA damage and repair (γ-H2AX and pChk1) and pathological complete response in TNBC patients treated with neoadjuvant chemotherapy (*N* = 66)

Biomarker	Pathological complete response		Chi2
	No	Yes	
	*N* (%)	*N* (%)	*p*-value
γ-H2AX^low^	18 (56.2)	14 (43.8)	0.009
γ-H2AX^high^	29 (85.3)	5 (14.7)	
pChk1^neg^	10 (55.6)	8 (44.4)	0.085
pChk1^pos^	37 (77.1)	11 (22.9)	

**Table 3:** Uni and multivariate logistic regression models of patient- and disease-related features and pathological complete response (*N*:66)

		Univariate logistic regression model ^(*)^		Multivariate logistic regression model ^(*)^	
		OR (95% CI)	*p*-value	OR (95% CI)	*p*-value
**Age**	**>48.4 vs ≤48.4**	4.13 (1.27–13.37)	0.018	6.15 (1.49– 25.44)	0.012
**Stage**	**III vs II**	0.81 (0.26–2.54)	0.723	1.23 (0.29–5.14)	0.780
**Grade**	**III vs I-II**	1.17 (0.40–3.46)	0.774	1.32 (0.34– 5.07)	0.687
**CT**	**Conc vs Seq**	Not applicable		Not applicable	
**Ki-67**	**≥60 vs <60**	0.29 (0.09–0.89)	0.030	0.31 (0.08–1.17)	0.084
**γ-H2AX**	**high vs low**	4.51 (1.39–14.66)	0.012	5.07 (1.28–20.09)	0.021
**pChk1**	**pos vs neg**	2.69 (0.85–8.48)	0.091	2.65 (0.63–11.19)	0.184

(*) Type of chemotherapy (concomitant vs sequential) was not included in uni- and multivariate models given that no pCRs were seen in patients treated with a concomitant schedule. Abbreviations, CT: chemotherapy; Conc: concomitant; Seq: sequential.

**Table 4:** Uni and multivariate logistic regression models of patient- and disease-related features and pathological complete response after removal of 13 patients whose hormone receptor status changed during neoadjuvant chemotherapy (*N*:53)

		Univariate logistic regression model ^(*)^		Multivariate logistic regression model ^(*)^	
		OR (95% CI)	*p*-value	OR (95% CI)	*p*-value
**Age**	**>48.4 vs ≤48.4**	3.78 (1.02–14.06)	0.047	4.65 (0.99–21.87)	0.052
**Stage**	**III vs II**	0.70 (0.19–2.63)	0.597	1.78 (0.30–10.43)	0.521
**Grade**	**III vs I-II**	1.08 (0.33–3.58)	0.899	1.46 (0.31- 6.84)	0.627
**CT**	**Conc vs Seq**	Not applicable		Not applicable	
**Ki-67**	**≥60 vs <60**	0.35 (0.10–1.19)	0.092	0.25 (0.05–1.19)	0.082
**γ-H2AX**	**high vs low**	4.71 (1.26–17.66)	0.021	7.07 (1.39–36.02)	0.018
**pChk1**	**pos vs neg**	1.40 (0.38–5.10)	0.610	1.10 (0.21–5.67)	0.909

(*) Type of chemotherapy (concomitant vs sequential) was not included in uni- and multivariate models given that no pCRs were seen in patients treated with a concomitant schedule. Abbreviations, CT: chemotherapy; Conc: concomitant; Seq: sequential.

## DISCUSSION

In the present study, we investigated the predictive ability of γ-H2AX and pChk1 expression in TNBC patients treated with NACT. To our knowledge, this is the first study pointing on these DNA damage and repair biomarkers as candidate predictive factors in TNBC. Overall, we observed a significant association between elevated levels of γ-H2AX and reduced pCR rate, whereas a similar association did not emerge for pChk1. We also observed a significant reduction in γ-H2AX levels when comparing primary and residual cancers. Considering the retrospective nature, in our opinion this study has some important strengths.

The neoadjuvant setting is ideal when the scope is the identification and development of predictive biomarkers. This is related to the short time span required to obtain efficacy data, the association between pCR and long-term survival outcomes, and the availability of pre- and post-treatment tumor tissues suitable for molecular analyses, at least for non-responders [4].

The logic behind this study was to investigate two cooperating DNA repair avenues, representing master regulators of the DDR machinery. The increased therapeutic resistance observed in tumors characterized by high levels of γ-H2AX raised the hypothesis that the ATM-Chk2 pathway is crucial for initiating DNA repair in TNBC cells.

Even though our data did not support a predictive role for pChk1 in TNBC, in our opinion the G_2_/M checkpoint should not be underestimated for different reasons. First, TP53 mutations are extremely common in TNBC [18]. p53-defective tumors are known to be extremely dependent on G_2_/M checkpoint activation to arrest the cell cycle and initiate DNA repair upon exposure to chemotherapy [17]. This form of “addiction” might therefore be exploited to look at potential G_2_/M checkpoint-associated biomarkers. Second, the use of carboplatin in the neoadjuvant setting has been recently found to achieve a greater rate of pCR in TNBC patients [19, 20], and platinum compounds represented the preferred partners for the development of Chk1 and Wee1 antagonists [17]. Thus, the impact of G_2_/M checkpoint-related molecular determinants on therapeutic outcomes in TNBC patients treated with carboplatin deserve to be investigated. Nonetheless, the involvement of the ATR-Chk1 and ATM-Chk2 pathways in the intra-S and G_2_/M checkpoints, and the connection between Chk1 and the spindle checkpoint, raised the hypothesis that their activation may confer chemoresistant features independently on the chemotherapy regimen used [17]. Overall, the lack of association between Chk1 and pCR, and the observation that ∼15% of patients whose tumors displayed elevated γ-H2AX levels experienced a pCR, encouraged us to initiate a more comprehensive analysis. To this end, our strategy for the development of a DDR signature envisions: i) The combined assessment of key components of the ATR-Chk1 and ATM-Chk2 pathways, e.g. pATM, pChk2, pATR, pWee1, pRPA32, together with genetic alterations that activate the DDR cascade, such as *TP53* mutations and *MYC* amplification [17], ii) Deeper characterization of the heterogeneity of TNBC, with a specific focus on the basal-like subtype, together with the assessment of androgen receptor expression (luminal androgen receptor subtype) given its potential as therapeutic target [21–23], and iii) The evaluation of multiple clinical outcomes, even including disease-free and overall survival. This second step will be instrumental for our prospective validation efforts. Moreover, the suggestion for an association between pChk1 and pCR in the subgroup of tumors that underwent a conversion in hormone receptor status was hypothesis-generating, and prompted us to undertake DDR analysis in luminal-type BC.

A further point that deserves mention relates to the analysis of residual disease. We would have expected an increase in γ-H2AX levels, as a consequence of the accumulation of DSBs following chemotherapy. Conversely, an opposite phenomenon was recorded. We can speculate that NACT operated an enrichment for slowly-cycling, chemotherapy-resistant cancer stem cells (CSCs) [24–27]. Considering that a series of studies, though retrospective yet, connected CSC-related endpoints with poorer survival outcomes [28], we envision that changes in γ-H2AX levels between pre- and post-NACT tissues might affect survival outcomes. An *ad hoc* study was designed to test this hypothesis.

In conclusion, γ-H2AX expression showed ability to foresee pCR in TNBC patients treated with anthracycline-taxane-based NACT. The results herein presented support the concept that DDR-related endpoints deserve further studies in TNBC.

## MATERIALS AND METHODS

This retrospective study has been conducted in accordance with the ethical standards and according to the Declaration of Helsinki and according to national and international guidelines and has been approved by the Ethic Committee of “Regina Elena” National Cancer Institute of Rome, the coordinating centre. Written informed consents were obtained before chemotherapy. Sixty-six patients treated with NACT were included in this retrospective analysis. Patients were considered eligible if the treatment was completed, data on clinical-pathological features were available, and tumors did not show HER2 overexpression/amplification according to ASCO-CAP guidelines. Concerning the expression of the estrogen receptor (ER) and progesterone receptor (PgR), 53 patients had TNBC in both diagnostic biopsies and in residual cancers when present, whereas 13 tumors switched their hormone-receptor status from weak positivity (ER or PgR ≤ 10%) in diagnostic biopsies to negativity in surgical samples (N: 10) or vice versa (N: 3). These patients were included based on the clinical plausibility of a basal-like molecular portrait, considering that up to 20% of basal-like cancers are not “pure” TNBC and express the ER [1]. Analyses were initially run in the entire cohort, and then repeated upon removal of these 13 samples. All patients had received anthracycline-taxane-based chemotherapy, either according to a concomitant or sequential approach. Of the 10 patients treated with concomitant chemotherapy, 9 received epirubicin 80 mg/m^2^ plus docetaxel 80 mg/m^2^ administered intravenously (IV) on day 1 every 3 weeks for four cycles, and 1 patient epirubicin 75 mg/m^2^ plus docetaxel 75 mg/m^2^ plus cyclophosphamide 500 mg/m^2^ IV on day 1 every 3 weeks for six cycles. In the 56 patients treated with sequential chemotherapy, epirubicin was given at 90 mg/m^2^ on day 1 every two weeks or 100–120 mg/m^2^ on day 1 every three weeks in 29 and 27 patients, respectively. In these patients, epirubicin was administered in association with cyclophosphamide 600 mg/m^2^ IV for four cycles, followed by docetaxel 100 mg/m^2^ IV on day 1 every 3 weeks for four cycles. pCR was defined as no residual invasive tumor in both breast and axilla, irrespective of the presence of ductal carcinoma *in situ* (ypT0/is ypN0), and was assessed by local pathologists. The immunohistochemical assessment of γ-H2AX and pChk1 was performed in formalin-fixed paraffin-embedded tissues using the anti-phospho-H2AX (Ser139) (clone JBW301) mouse monoclonal antibody (MAb) (Upstate) at the dilution of 1:500, and the anti-phospho-Chk1 (Ser345) (clone 133D3) rabbit MAb (Cell Signaling) at the dilution of 1:150. γ-H2AX expression was considered as the percentage of nuclear-expressing tumor cells and analyzed as a categorical variable, using the median score of all tumors to define high and low expressing samples (γ-H2AX^low^ and γ-H2AX^high^). pChk1 was graded based on nuclear staining intensity (0: negative, 1+: weak, 2+: moderate, 3+: strong), and it was considered as negative (0: pChk1^neg^) or positive (1–3: pChk1^pos^). Two investigators (ADB and CE) blinded to the outcome independently evaluated immunoreactivity. A third investigator (MM) reviewed discordant cases.

### Statistical analysis

Clinical, pathological and molecular features were descriptively characterized for all the patients included in the present analysis. Continuous variables were reported as medians and ranges, and categorical variables were expressed by frequencies and percentage values. The Pearson's Chi-squared test of independence (2-tailed) and the Fisher Exact test were used to assess the relationship between categorical variables. Univariate logistic regression models were used to identify variables impacting treatment outcome, and multivariate logistic regression models were built by including variables significant at the univariate assessment or based on their clinical or biological plausibility in influencing pCR. Internal validation was conducted through a re-sampling procedure without replacement in order to estimate the risk of an overfitted model [29]. By randomly removing ∼20% of the original sample, one hundred less-powered datasets were created and, for each simulation, the multivariate logistic regression model was carried out. For each simulation we calculated the Cohen's Kappa coefficient. The replication rate was also calculated. The Wilcoxon test was used to evaluate pre- and post-NACT changes in γ-H2AX and Ki-67. We considered statistically significant *p* values less than 0.05. Statistical analyses were carried out using SPSS software (SPSS version 21, SPSS Inc., Chicago, IL, USA).
